# Extracting mechanical quality factor and eliminating feedthrough using harmonics of thermal-piezoresistive micromechanical resonators

**DOI:** 10.1038/s41378-025-00869-8

**Published:** 2025-02-20

**Authors:** Geer Teng, Chenhao Yang, Aojie Quan, Chengxin Li, Haojie Li, Yuxuan Cheng, Honglong Chang, Michael Kraft, Hemin Zhang

**Affiliations:** 1 MOE Key Laboratory of Micro and Nano Systems for Aerospace, School of Mechanical Engineering, Northwestern Polytechnical University, 710072 Xi’an, China; 2 Micro and Nano Systems-ESAT, KU Leuven, Leuven, Belgium; 3 Key Laboratory of Scale Manufacturing Technologies for High-Performance MEMS Chips of Zhejiang Province, Key Laboratory of Optical Microsystems and Application Technologies of Ningbo City, Ningbo Institute of Northwestern Polytechnical University, 315103 Ningbo Zhejiang, China

**Keywords:** Electrical and electronic engineering, NEMS

## Abstract

Thermal-actuation and piezoresistive-detection effects have been employed to pump the effective quality factor of MEMS resonators, targeting simple self-oscillation and better sensing performance in the air. However, the ratio of the pumped effective quality factor to the inherent mechanical quality factor, crucial for characterizing the amplification, is hard to obtain. The main difficulty stems from hidden Lorentz peaks caused by feedthrough effects and the pump effect once the current is applied. In this paper, we demonstrated the presence of high-order harmonic components in the output of thermal-piezoresistive resonators when the oscillation amplitude is sufficiently large. By utilizing second-order harmonics, we achieved the improvement in signal-to-bias ratio of, 20.85 dB compared to that without feedthrough cancellation and 9.67 dB compared to that using a de-embedded method when the bias current is 6.20 mA. Furthermore, the inherent mechanical quality factor is extracted at a low current of 1.8 mA with a value of 5800 using the second-order harmonics, and a nearly two orders of magnitude enhancement in Q factor can be obtained before entering the self-oscillation regime. An amplitude bias instability as good as 55 ppm and a frequency bias instability as good as 10 ppb are realized in the nonlinear operation regime with a pumped effective quality factor of 576k. The paper contributes to the fundamental understanding of the Q pump effect together with harmonic analysis of the thermal-piezoresistive resonators and also pushes forward the development of low-power consumption self-oscillation resonant sensors.

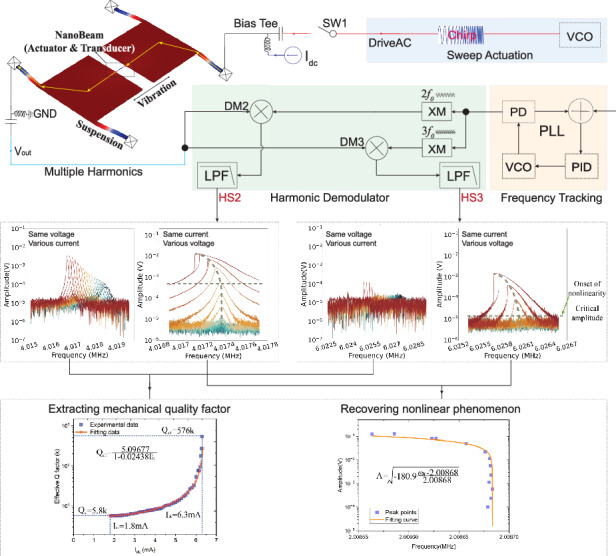

## Introduction

Microelectromechanical (MEMS) resonators have been widely employed in various sensing applications, such as mass sensors^1–4^, resonant accelerometers^5,6^, as well as gyroscopes. The mechanical quality (${Q}_{{\rm {m}}}$) factor of resonators is a measure of the energy dissipation during a specific working cycle and a crucial parameter to improve the signal-to-noise ratio (SNR) of force sensors, phase noise of oscillators, and noise rejection of filters^7^. To improve ${Q}_{{\rm {m}}}$, either intrinsic dissipation, like thermoelastic losses, or extrinsic damping like clamping losses, should be reduced^8^. In contrast, the effective quality factor (${Q}_{{{\rm {eff}}}}$) can be manipulated by feeding energy to the resonator without modifying the intrinsic or extrinsic dissipations. External feedback control has become the most classical method of ${Q}_{{{\rm {eff}}}}$ tuning^9^.

In the past decade, thermal-actuation and piezoresistive-detection mechanisms have been demonstrated as a beneficial method to increase ${Q}_{{{\rm {eff}}}}$ of resonators working in ambient environments^10–18^. Principally, the nanobeam expands once a bias DC current is applied through it, causing defections due to Joule heating power. Consequently, the energy delivered by the input current compensates for the dissipations of the resonator caused by mechanical motion, resulting in a significant improvement on ${Q}_{{{\rm {eff}}}}$. When thermal compensation is sufficient, self-oscillation can be achieved, even in air-operating conditions^19^.

The frequency response fitting and ringdown methods are the two classical mechanisms^7,20^ to extract ${Q}_{{{\rm {eff}}}}$. However, these methods are not feasible in measuring ${Q}_{{\rm {m}}}$ of thermal-piezoresistive resonators (TPRs), because once the mandatory bias DC current is applied, Q will be pumped immediately. Another barrier is that, in contrast to MEMS capacitive resonators designed with differential sensing electrodes to eliminate the feedthrough effect, the outputs of TPRs always have a significant feedthrough signal component, due to no feasible differential sensing method for feedthrough cancellation^21^. As a result, the voltage output with feedthrough sometimes cannot accurately characterize the motion of a TPR, especially in cases with low bias current the coupling actuation AC signal is too large to monitor the effective motional voltage of the resonators^22,23^. Even using the ringdown characterization method, the signal is severely affected by the feedthrough signal^24^. Consequently, in this case, ${Q}_{{\rm {m}}}$ cannot be correctly characterized by a measured frequency response and no previous research experimentally demonstrates how much ${Q}_{{{\rm {eff}}}}$ can be pumped. Furthermore, due to the feedthrough signal, Duffing nonlinear^25,26^ characteristics of the TPRs are difficult to be studied as the signal-to-bias ratio is too small for identifying the bifurcations^27–29^.

In this paper, we experimentally found that there are high-order (second-order and third-order) harmonic components in the output of TPRs when the oscillation amplitude is sufficiently high, especially in the Duffing nonlinear regime. With a specific actuation level, the amplitude of the second-order harmonic signal is about one order of magnitude lower than that of the signal at the natural frequency. Using these harmonic components, basic information, including vibrating frequency and nonlinear amplitude–frequency (A–f) effect, can be deduced and the feedthrough signal can be suppressed. On the other hand, benefiting from the feedthrough cancellation, Q factor can be calculated precisely, and it is found that the Q factor of a TPR cannot be pumped by the bias current if this current is at a low level, indicating the inherent ${Q}_{{\rm {m}}}$ of the TPR. Besides, we experimentally proved the pumping Q factor of TPRs beneficial for the resolution of oscillators. This paper provides an alternative method of characterizing the frequency response and *Q*-factor information of thermal-piezoresistive resonators with feedthrough elimination.

## Results

### Thermal actuation and piezoresistive detection

**Fig. 1:**
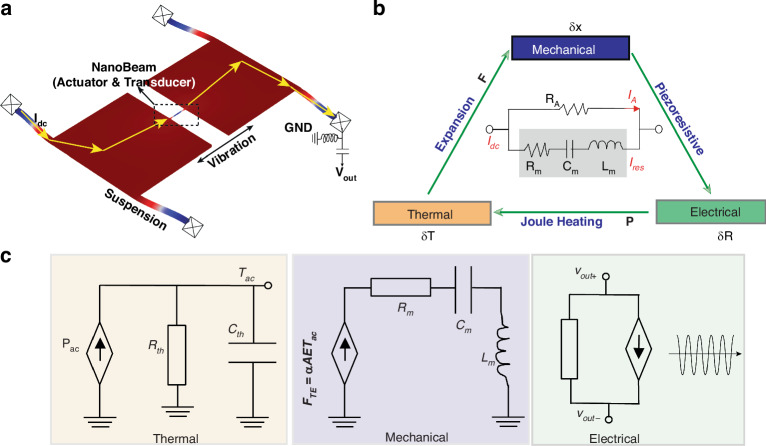
Schematic and model of the thermal-piezoresistive resonator. **a** Finite element analysis of the resonator indicating the in-plane vibration mode. **b** Multidisciplinary illustration of the thermal-piezoresistive principle. **c** Equivalent transfer circuits of the thermal-piezoresistive resonator

The proposed TPR is constructed with two movable plates supported by four suspension beams and connected by a nanobeam (Fig. 1a). The movement of a TPR results from the periodic thermal expansion and contraction of the nanobeam. The operating principle of a TPR involves several physical domains, combining thermal, mechanical, and electrical effects^11^ and is illustrated in Fig. 1b. A direct current (${I}_{{{\rm {dc}}}}$) flowing through the nanobeam expands the nanobeam with a resistance of *R*_0_ to a pre-expansion state due to the Joule heating power of P_0_ = I_dc_^2^R_0_. The additional harmonic actuation signal (*v*_ac_) causes periodic expansion and contraction of the nanobeam via Joule heating/cooling, with a power of ∆P ≈ v_ac_^2^/R_0_. Consequently, a sinusoidally variable resistance ($\delta {R}_{{{\rm {ac}}}}$) is generated due to the negative piezoresistive coefficient with the value of $\delta {R}_{{{\rm {ac}}}}$ dependent on the actuation voltage *v*_ac_. The plates are actuated by the nanobeam and oscillating with the same frequency if *v*_ac_ is not high enough to drive the TPR into the Duffing nonlinear regime. The nanobeam deflection is finally transferred to an alternating output voltage (${\rm{v}}_{{{\rm{out}}}}={\rm{I}}_{{{\rm {dc}}}}\cdot \delta {\rm{R}}_{{{\rm {ac}}}}$).

The nanobeam can be modeled as the parallel combination of its internal resistance *R*_A_ and the equivalent RLC circuit of the mechanical resonator, as shown in the inset of Fig. 1b. Energy transformations between different physical domains can be described by the equivalent circuits as illustrated in Fig. 1c. Detailed derivations of the transfer functions in each domain are provided in Supplementary Material Note 1. Simplifying the total transfer function relating output current to input voltage, we have the equivalent conductance of the resonator ${g}_{{\rm {m}}}$^12^$:$

1 $${H}_{{{\rm {total}}}}=\frac{{T}_{{{\rm {ac}}}}}{{v}_{{{\rm {ac}}}}}\cdot \frac{{X}_{{{\rm {th}}}}}{{T}_{{{\rm {ac}}}}}\cdot \frac{{i}_{{\rm {m}}}}{{X}_{{{\rm {th}}}}}={g}_{{\rm {m}}}\approx \frac{2\alpha E{Q}_{{\rm {m}}}{\pi }_{l}}{{\omega }_{0}{C}_{{{\rm {th}}}}}{I}_{{{\rm {dc}}}}^{2}$$

where ${T}_{{{\rm {ac}}}}$ is the temperature fluctuation, ${v}_{{{\rm {ac}}}}$ the input voltage, ${X}_{{{\rm {th}}}}$ the displacement fluctuation and ${i}_{{\rm {m}}}$ the output current. ${R}_{{{\rm {th}}}}$, ${C}_{{{\rm {th}}}}$ and $\alpha$ represent thermal resistance, thermal capacitance and thermal expansion coefficient of the actuating nanobeam. $E$ is the Young’s modulus, ${\omega }_{0}$ the natural frequency of the resonator and ${\pi }_{{\rm {l}}}$ the piezoresistive coefficient. Through the transfer functions of Joule heating and thermal expansion effects, the thermal expansion force can be written as

2 $${F}_{{{\rm {TE}}}}(s)=\alpha {T}_{{{\rm {ac}}}}{EA}=\alpha {KE}{\pi }_{{\mathcal{l}}}{R}_{{\rm {A}}}{X}_{{{\rm {th}}}}\frac{2{{I}_{{{\rm {dc}}}}}^{2}{R}_{{{\rm {th}}}}}{1+{R}_{{{\rm {th}}}}{C}_{{{\rm {th}}}}s}$$

where $K$ is the stiffness of the resonator. Specifically in the mechanical domain, the dynamic equations of the resonator can be expressed as

3 $${\ddot{X}}_{{{\rm {th}}}}+\frac{c}{M}{\dot{X}}_{{{\rm {th}}}}+\frac{K}{M}{X}_{{{\rm {th}}}}=\frac{{F}_{{{\rm {TE}}}}}{M}={{I}_{{{\rm {dc}}}}}^{2}{{\omega }_{0}}^{2}\chi$$

4 $$\chi =\frac{2\alpha E{\pi }_{{\mathcal{l}}}{R}_{{\rm {A}}}}{{\left({\omega }_{0}{C}_{{{\rm {th}}}}\right)}^{2}}\left(\frac{1}{{R}_{{{\rm {th}}}}}-j{\omega }_{0}{C}_{{{\rm {th}}}}\right)$$

where $M$ is the effective mass, $c$ the damping. The effective Q can be derived from the imaginary part with ${X}_{{{\rm {th}}}}={x}_{0}{{\rm {e}}}^{j{\omega }_{0}t}$:

5 $${Q}_{{{\rm {eff}}}}=\frac{{Q}_{{\rm {m}}}}{1+{R}_{{\rm {A}}}{g}_{{\rm {m}}}}=\frac{{Q}_{{\rm {m}}}}{1+{\rm{{\Upsilon }}}{I}_{{{\rm {dc}}}}^{2}},{\rm{{\Upsilon }}}={R}_{{\rm {A}}}\frac{2\alpha E{Q}_{{\rm {m}}}{\pi }_{{\mathcal{l}}}}{{\omega }_{0}{C}_{{{\rm {th}}}}}$$

As the piezoresistive coefficient ${\pi }_{l}$ is negative, in particular for resonators with relatively high frequency, there are specific values for ${I}_{{{\rm {dc}}}}^{2}$ keeping ${R}_{{\rm {A}}}{g}_{{\rm {m}}}\subset [-\mathrm{1,0}]$, and pumping the effective quality factor with currents increasing.

### Device fabrication and measurement setup

**Fig. 2:**
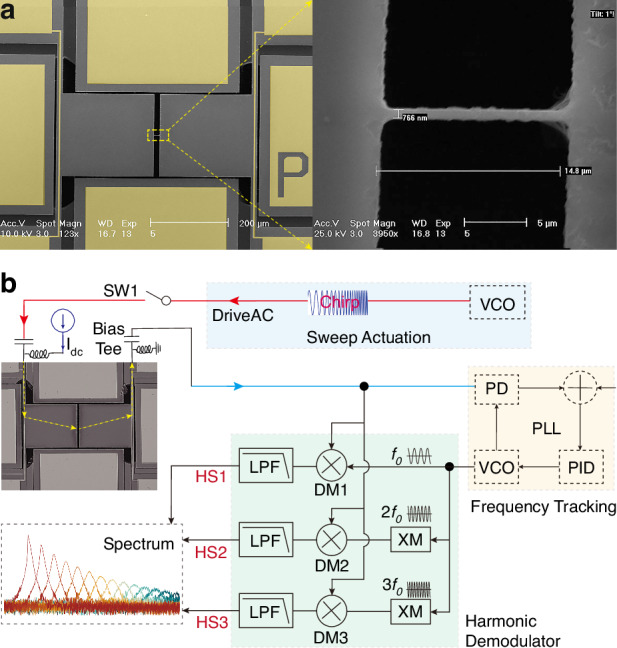
Device images and experimental implementation diagram. **a** Optical images of the fabricated thermal-piezoresistive resonator and the nanobeam. **b** Electrical characterization setup where DM is the demodulator, XM the multiplexer, LPF the low pass filter, HS the harmonic signal, SW the switch, and VCO the voltage-controlled oscillator

A 10 µm-thickness silicon-on-insulator (SOI) wafer was used to fabricate the device based on a dicing-free fabrication process^30^. The nanobeam is designed with a width of 1 µm. Due to over-etching, the fabricated width of the nanobeam is measured ~760 nm. An optical image of the resonator is shown in Fig. 2a. The device layer is n-doped with a dopant concentration of 5 × 10^18^ cm^–3^, resulting in a negative piezoresistive coefficient. The nanobeam acts simultaneously as an actuator and a displacement sensor.

An open-loop setup, as shown in Fig. 2b was conducted to explore the input-output responses of the TPR. A chirp signal was generated by the internal digital oscillator of a Zurich lock-in amplifier (HF2LI) to actuate the TPR with a frequency of ${f}_{{\rm {d}}}$. Three demodulators were utilized to demodulate the output signal with the frequency of ${f}_{{\rm {d}}}$, ${2f}_{{\rm {d}}}$ and $3{f}_{{\rm {d}}}$, to extract the harmonic components of the output. All functions were realized with the lock-in amplifier, excluding the bias DC current which was supplied by a power meter.

### Harmonics and feedthrough suppression

In the open-loop measurements, a chirp signal around the natural frequency is applied through a bias tee to actuate the resonator. This actuation signal is also directly coupled to the output and is mixed with the motional signal, which causes a feedthrough component to the output.

**Fig. 3:**
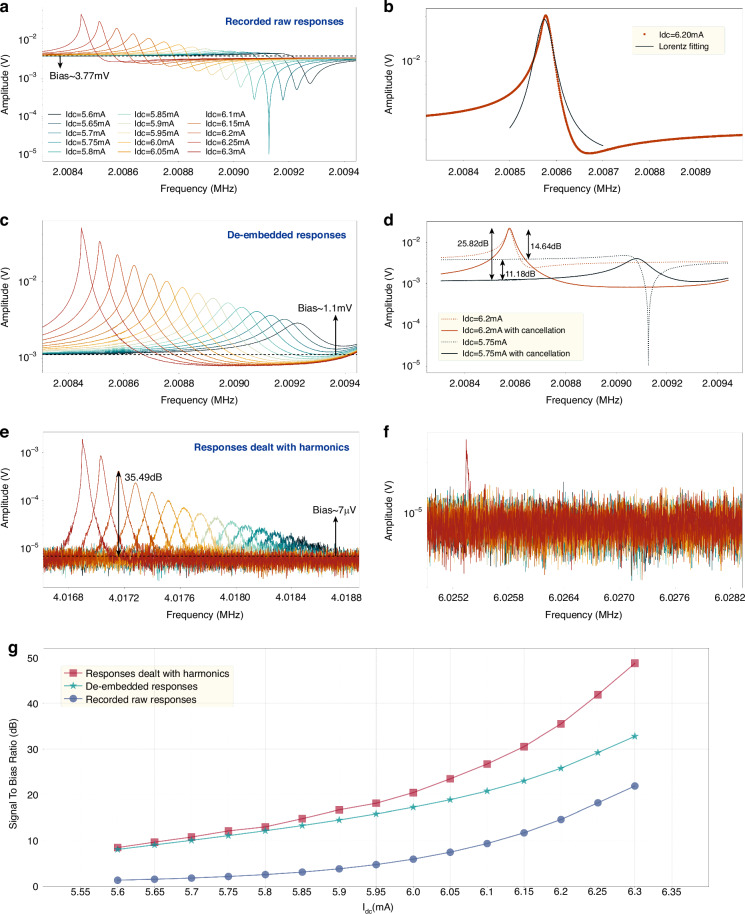
Frequency responses under different handlings and corresponding signal-to-bias ratios. **a** Recorded raw output amplitude–frequency responses of the TPR actuated with various currents. **b** Lorentz fitting curve of the frequency response with bias current equaling to 6.20 mA. **c** Processed amplitude-frequency spectrum with the de-embedded feedthrough cancellation method. **d** Comparison between the responses with and without the de-embedded feedthrough cancellation when bias current is 6.20 and 5.75 mA. Obtained frequency responses of the TPR using the second- (**e**) and the third- (**f**) order harmonic components to demodulate the mixing signals. The nonlinearity begins at the current of 6.30 mA and the corresponding signal generates in the response demodulated with the third-order harmonic. **g** The relationships between the signal-to-bias ratio and the bias current of frequency responses recorded, calculated with the de-embedded method, and dealt with the second-order harmonics

To characterize the resonance frequency and effective quality factor ${Q}_{{{\rm {eff}}}}$ of the device, open-loop frequency sweeps were conducted with varying direct current ${I}_{{{\rm {dc}}}}$. We took the bias currents striding regimes from attenuation to amplification, all below the threshold of self-oscillation, as illustrated in Fig. 3a. The −3 dB bandwidth method was employed to estimate ${Q}_{{{\rm {eff}}}}$ and the response shows a good match with the Lorentz fitting curve, as shown in Fig. 3b. A significant observation is that using the Lorentz fitting function or −3 dB method to extract Q is only feasible when the bias current is moderate. There is significant nonlinear hysteresis when the bias current is sufficiently high. When the current is very low, the Lorentz peak is insignificant or even disappears whereas an anti-resonance^31^ is observed, which is attributed to the feedthrough directly coupled from the lock-in amplifier.

Notably, unobvious signal-to-bias ratios are commonly visible in recorded raw frequency responses, which are demodulated using the same frequency (${f}_{{\rm {d}}}$) as the drive AC signal in Fig. 3a. Significant anti-resonance peaks in low current region also can be observed, which indicates feedthrough effects. To achieve pure output by eliminating the mixed feedthrough signal, a de-embedded feedthrough cancellation method has been proposed^32^, wherein the extracted signal is used to subtract from the input reference signal. This reference signal includes the coupling from the actuation AC but lacks any motional signal. In our device, this reference was achieved by measuring the output while applying direct current *I*_dc_ = 0 mA. Details of this method are put in Supplementary Material Note 2.

The analysis of Fig. 3c and a suggests that frequency responses, following the removal of feedthrough, demonstrate consistent amplitudes without any signs of anti-resonance. The signal-to-bias ratio is improved by 11.18 dB, from 14.64 to 25.82 dB when the bias current ${I}_{{{\rm {dc}}}}$ is 6.20 mA, as shown in Fig. 3d. Nevertheless, this method requires extra calculations and offline processing, introducing added complexity.

In our experiments, we found that there exist clean outputs without the influence of feedthrough if the signal is demodulated by two or three times of the drive frequency. The second-order harmonic can be explained by the thermal actuation power which is expressed as the combination of ${V}_{{{\rm {dc}}}}$ and ${V}_{{{\rm {ac}}}}$:

6 $$\begin{array}{l}P=\frac{({{V}_{{{\rm {ac}}}}\,{\rm {cos}}\, 2\pi {f}_{{\rm {d}}}t+{V}_{{{\rm {dc}}}}})^{2}}{{R}_{{\rm {A}}}}=\frac{{{V}_{{{\rm {ac}}}}}^{2}}{{R}_{{\rm {A}}}}{\rm {cos}}^{2}\,2\pi {f}_{{\rm {d}}}t\\\qquad+\,2\frac{{V}_{{{\rm {ac}}}}{V}_{{{\rm {dc}}}}}{{R}_{{\rm {A}}}}{\rm {cos}}\, 2\pi {f}_{{\rm {d}}}t+\frac{{{V}_{{{\rm {dc}}}}}^{2}}{{R}_{{\rm {A}}}}\end{array}$$

There is a component at the frequency of ${f}_{{\rm {d}}}$, inducing the corresponding harmonic signal at ${f}_{{\rm {d}}}$ as well as ${2f}_{{\rm {d}}}$. The demodulated signal with the frequency of ${2f}_{{\rm {d}}}$ is shown in Fig. 3e, indicating a bias voltage of 7 µV and a signal-to-bias ratio ~35.49, 9.67 dB higher than that of the signal using the de-embedded feedthrough cancellation method when the bias current ${I}_{{{\rm {dc}}}}$ is 6.20 mA. In addition, in cases of low DC current, there is no significant anti-resonance, while clear Lorentz peaks are visible. In such a situation, we can measure the Q factor using the −3 dB method with frequency responses.

Also, the signal-to-bias ratios of raw responses, de-embedded responses and responses dealt with harmonics are exhibited in Fig. 3g, illustrating the optimization of feedthrough suppression with harmonics. It is visible that the signal-to-bias ratio enhances as the bias current increases and the ratio of responses dealt with harmonics is commonly higher than that of de-embedded responses. Moreover, the optimization with harmonics is more notable at high currents.

**Fig. 4:**
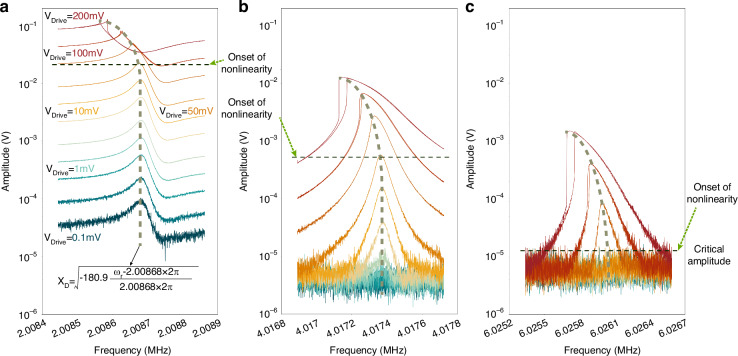
Frequency responses with various drive AC. Frequency responses of the TPR with a DC current of *I*_dc_ = 6.10 mA, while the actuation ac level is variable from 0.1 to 200 mV, and the signal is demodulated with the frequency of ${f}_{{\rm {d}}}$ (**a**), ${2f}_{{\rm {d}}}$ (**b**) and ${3f}_{{\rm {d}}}$ (**c**). Nonlinearity occurs at the drive AC voltage of 50 mV

In order to have a better view of the influence of the nonlinearity to the harmonic signal, we recorded the frequency responses of the TPR with variable drive AC voltages while the bias DC current was fixed at ${I}_{{dc}}$ = 6.10 mA. A clear amplitude-frequency (*A*–*f*) backbone curve can be observed in Fig. 4a. A phenomenon is that the second-order (Fig. 4b) harmonic signal is observable in both the linear and nonlinear regimes, while the third-order (Fig. 4c) harmonic signal appears only in the nonlinear regime.

It is believed that the phenomenon is due to the nonlinearity of the device, particularly the Duffing nonlinearity in the mechanical domain. Evidence is that only in the case of the device operating in the nonlinear regime (e.g for *I*_dc_ = 6.30 mA) we can see the appearance of a harmonic signal at ${3f}_{{\rm {d}}}$, as shown in Fig. 3f. Theoretically, the thermal actuation force when concerning Duffing nonlinearity can be rewritten as

7 $${F}_{{{\rm {TE}}}}(s)=\alpha E{\pi }_{{\mathcal{l}}}{R}_{{\rm {A}}}\frac{2{{I}_{{{\rm {dc}}}}}^{2}{R}_{{{\rm {th}}}}}{1+{R}_{{{\rm {th}}}}{C}_{{{\rm {th}}}}s}(K{X}_{{{\rm {th}}}}+{K}_{3}{{X}_{{{\rm {th}}}}}^{3})$$

where ${K}_{3}$ represents the coefficient of Duffing nonlinearity. Even if we assume that the simple wave with natural frequency is primary in the displacement, there is still a term of ${{X}_{{{\rm {th}}}}}^{3}$ generating the signal with frequency of ${3f}_{{\rm {d}}}$.

Complete derivation and further explanation of nonlinearity are described in the Supplementary Material Note 3. With the multi-harmonic balance method to solve the dynamic equation considering nonlinear terms, the peak value of the amplitude ${X}_{{\rm {D}}}$ in Fig. 4a is obtained:

8 $${{X}_{{\rm {D}}}}^{2}\approx \frac{8K}{3{K}_{3}}\frac{{\omega }_{{\rm {r}}}-{\omega }_{0}}{{\omega }_{0}}\approx -180.9\frac{{\omega }_{{\rm {r}}}-2.00868\cdot 2\pi }{2.00868\cdot 2\pi }$$

The nonlinear coefficient ${K}_{3}$ is calculated to be $-7186\,{\rm {N/{m}}}^{3}$, using $K=M{{\omega }_{0}}^{2}$. The detailed parameters and amplitude–frequency curve are shown in Supplementary Material Fig. S2.

### Self-oscillation and mechanical *Q* extraction

Referring to the principle of thermal actuation, as the input DC current increases, the motion of the TPR experiences attenuation, amplification, and finally exhibits self-excited oscillation^16^ when the threshold is reached.

**Fig. 5:**
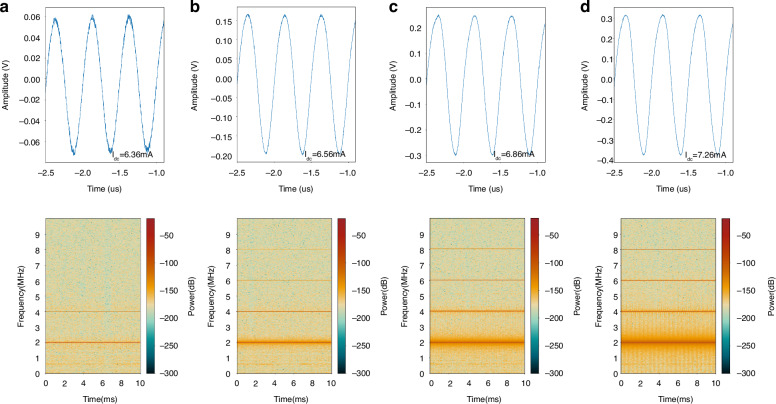
Self-oscillation analysis in the time domain. Self-oscillation amplitudes in the time domain and the corresponding spectrograms with various direct currents from 6.36 mA (**a**), 6.56 mA (**b**), 6.86 mA (**c**) to 7.26 mA (**d**). The distortion of the oscillation signal becomes more and more significant with the increasing of the bias current, and meanwhile, the high-order harmonics gradually appear

For the steady oscillation at the frequency of 2.008 MHz, with a sampling rate of 625 MHz at specific amplitudes, mixing harmonic signals are observed with the bias current ${I}_{{{\rm {dc}}}}$ varying from 6.36, 6.56, 6.86 to 7.26 mA. Using a short-time Fourier transform to deal with the mixing signals, the time–frequency diagrams can be obtained, and clear frequency characteristics are visible, as being recorded in Fig. 5. This demonstrates that high-order harmonics normally exist as long as the current reaches a certain value. A higher current creates more harmonics and more significant distortion.

**Fig. 6:**
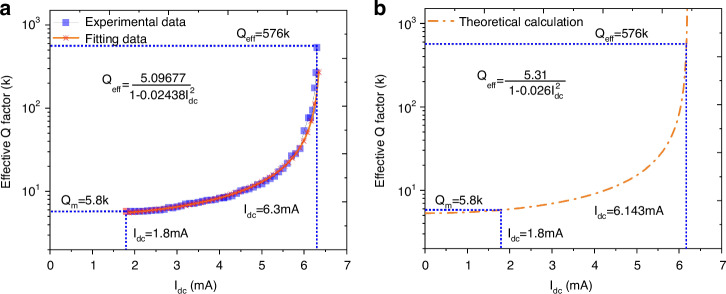
Experimental measurement and theoretical stimulation of Q_eff_ in thermal-piezoresistive pumping. Extracted effective Q factor and the fitting curve of interrelation between effective Q factor and current of the experiment (**a**) together with the theoretical calculation (**b**). Theoretical calculation is processed on the basis of Eq. (5) combining the extracted mechanical ${Q}_{{\rm {m}}} \sim 5.8k$, and the following settings: *π*_*l*_ = −50 × 10−11 Pa^−1^, *α* = 2.6 × 10^−6^ K^−1^, *E* = 169 GPa, ${\omega }_{0}=2\pi \times 2.008\,{\rm{MHz}}$, *ρ* = 0.0018 Ω cm, the length $L$ and cross area $A$ of nanobeam, respectively 14.8 and 0.76 µm × 10 µm, the density of mass *ρ*_m_ = 3 g cm^−3^, the specific heat capacity *c*_H_ = 800 J kg^−1^ K^−1^, *R*_A_ = *ρ*_m_*L*/*A* and *C*_th_ = *c*_H_ρ_m_*LA*

Although the high-order harmonic induces distortion, it also suppresses the feedthrough signal as mentioned in the section “Harmonics and feedthrough suppression”. The Lorentz peaks can be recovered even with a very low bias current. Therefore, it is possible to extract the effective quality factor ${Q}_{{{\rm {eff}}}}$ by the −3 dB method anyway. The ${Q}_{{{\rm {eff}}}}$ of the TPR, extracted using second-order harmonics (as shown in Fig. 3e), is illustrated in Fig. 6a for bias currents ranging from 1.80 to 6.3 mA. It is observable that ${Q}_{{{\rm {eff}}}}$ will no longer decrease when the bias current is lower than 1.80 mA, which indicates a convergence phenomenon. In other words, this implies that ${Q}_{{{\rm {eff}}}}$ is being pumped only with a current >1.80 mA. Thus, the ${Q}_{{{\rm {eff}}}}$ with ${I}_{{{\rm {dc}}}}=$1.80 mA can be considered as the mechanical ${Q}_{{\rm {m}}}$ ~ 5.8k as shown in Fig. 6a. In addition, the relation between ${Q}_{{{\rm {eff}}}}$ and the current can be fitted as

9 $${Q}_{{{\rm {eff}}}}\approx \frac{5.09677}{1-0.02438{{I}_{{{\rm {dc}}}}}^{2}}$$

According to the extracted ${Q}_{{{\rm {eff}}}}$ ~ 576k where the resonator goes into self-oscillation, the quality factor has been pumped by a factor of 99.3, nearly two orders of magnitude. The theoretical predication of ${Q}_{{{\rm {eff}}}}$ versus ${I}_{{{\rm {dc}}}}$ can be found in Fig. 6b, matching the experimental results quite well. The disparity in ${I}_{{{\rm {dc}}}}$ when the pumped ${Q}_{{{\rm {eff}}}}$ approaches 576k is attributed to the differences in the simulation settings and the actual properties of the material.

### Amplitude and frequency stability under pumped Q

The above results have demonstrated the effective pump of ${Q}_{{{\rm {eff}}}}$ in thermal-piezoresistive resonators. However, it is not clear whether such a pump is beneficial to the amplitude and frequency resolution.

To clarify the influences of the enhanced Q factor on the resolution and stability of the amplitude and frequency, a closed loop configuration was implemented using the lock-in amplifier, in which the drive AC voltages were set to 1 and 300 mV, representing the linear and nonlinear operation regimes. We calculated the corresponding noise density and Allan deviations of the amplitude as well as frequency to evaluate the influence of pumped ${Q}_{{{\rm {eff}}}}$ on the performance of the TPR.

**Fig. 7:**
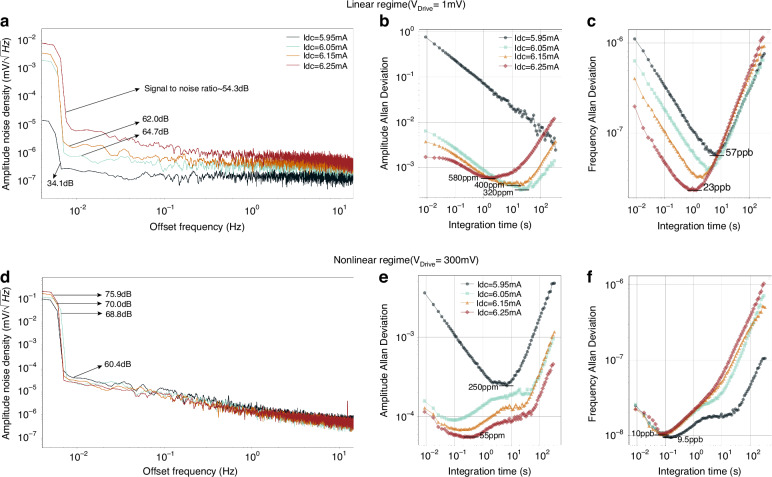
Analysis of the frequency and amplitude resolution in linear and nonlinear operation regimes. With a drive AC voltage of 1 mV, amplitude noise density (**a**), amplitude Allan deviation (**b**) and frequency Allan deviation (**c**) with different direct currents. Amplitude noise density (**d**) at the drive voltage of 300 mV and amplitude Allan deviation (**e**) as well as frequency Allan deviation (**f**) at the corresponding bias situations

In the linear operation regime, with the current increasing and Q factor being pumped, the amplitude noise density roughly decreases while the signal-to-noise ratio generally increases as shown in Fig. 7a. With the bias current *I*_dc_ changing from 6.05 to 6.25 mA, the bias instability of the amplitude is enlarged from 320, 400 to 580 ppm as indicated in Fig. 7b.

A much higher drive AC voltage of 300 mV drives the resonator into the nonlinear operation regime. The relevant amplitude noise density of various currents is shown in Fig. 7d. The signal-to-noise ratio slightly increases with the current boosting and is generally higher than that in a linear regime. Also, the amplitude bias instability is significantly improved with increasing currents, from 250 ppm@*I*_dc_ = 5.95 mA, to 55 ppm@*I*_dc_ = 6.25 mA, as shown in Fig. 7e.

For the frequency, in the linear regime, the bias instability varies from 57 ppb@*I*_dc_ = 5.95 mA to 23 ppb@*I*_dc_ = 6.25 mA as indicated by Fig. 7c, whereas it keeps almost unchanged in the nonlinear operation regime as shown in Fig. 7f. Though the direct currents and drive voltages vary, frequency bias instability has little difference and keeps generally below 10ppb, exhibiting very high stability in short integration time term of 0.1 s.

As the effective quality factor has been pumped from *I*_dc_ = 5.95 mA to *I*_dc_ = 6.25 mA, the pumped ${Q}_{{{\rm {eff}}}}$ is beneficial to improve the amplitude stability both in the linear and nonlinear operation regimes, while the frequency stability can only be improved in the linear operation regime.

## Discussion

As a promising transduction method, thermal actuation and piezoresistive detection offer an attractive avenue for the development of simple, self-sustained oscillators with low power consumption, while eliminating the need for vacuum packaging. The Joule heating energy generated by the bias current serves to offset the energy consumed by the mechanical motion of the resonators. Consequently, this mechanism enhances the effective quality factor (${Q}_{{{\rm {eff}}}}$) which, with an increase in bias current, can reach levels sufficient to sustain self-oscillation. However, the quantification factor of the pumped ${Q}_{{{\rm {eff}}}}$ compared to the mechanical Q remains unexplored. This is due to the masking effect of high feedthrough signals on the observable Lorentz peaks at low bias currents, which hinders the precise extraction of the mechanical quality factor.

Our experimental findings in this study highlight the presence of high-order harmonics that provide crucial insights. The second-order harmonics are linked to quadratic power generation, while the third-order harmonics are presumed to arise from Duffing nonlinearity. Through frequency sweeps of these harmonics, we achieve a notable 20.85 dB enhancement in signal-to-bias ratio at specific bias configuration, facilitating easy and precise extraction of the quality factor regardless of the bias current value. Remarkably, our results indicate a prominent amplification of the quality factor by a factor of 99.3 before the resonator enters into the self-oscillation regime. Although the method using harmonics to eliminate feedthrough has never been applied to capacitive resonators^33,34^, we extend this application and achieve further nonlinearity derivation with multi-harmonics.

Subsequent performance analysis shows that, for the thermal-piezoresistive resonators, the pumped effective quality factor enhances the amplitude stability both in the linear and nonlinear operation regimes, whereas the frequency stability can only be improved in the linear operation regime. An amplitude bias instability as good as 55 ppm and a frequency bias instability as good as 10 ppb are realized in the nonlinear operation regime with a bias current of *I*_dc_ = 6.25 mA. This improvement differs from the traditional consideration that stability and resolution are defined significantly by the mechanical quality factor but not the effective quality factor. A deep study should be developed in the future to push forward the fundamental understanding of the quality factor pumping effect based on thermal-actuation and piezoresistive-detection methods, and the applications of thermal-piezoresistive resonators in more engineering fields, like inertial sensing and time references.

## Supplementary information

Supplementary Material
