# Supplementary material for: Extracting mechanical quality factor and eliminating feedthrough using harmonics of thermal-piezoresistive micromechanical resonators
Source: Microsyst Nanoeng. 2025 Feb 20;11:30. doi: 10.1038/s41378-025-00869-8 (PMC11842816; doi:10.1038/s41378-025-00869-8)
Supplement: Supplementary file 1 — Supplementary Material [file 41378_2025_869_MOESM1_ESM.docx]

Supplementary Material

For

Extracting Mechanical Quality Factor and Eliminating Feedthrough Using Harmonics of Thermal-Piezoresistive Micromechanical Resonators

Geer Teng^1†^, Chenhao Yang^1†^, Aojie Quan^3†^, Chengxin Li^3^, Haojie Li^1^, Yuxuan Cheng^1^, Honglong Chang^1^, Michael Kraft^3^and Hemin Zhang^1,2^

^1^MOE Key Laboratory of Micro and Nano Systems for Aerospace, School of Mechanical Engineering, Northwestern Polytechnical University, Xi’an 710072, Chinna,

^2^Ningbo Institute of Northwestern Polytechnical University, Northwestern Polytechnical University, Xi’an 710072, China.

^3^Micro and Nano Systems - ESAT, KU Leuven, Leuven, Belgium

Email of all authors: Hemin Zhang is the corresponding author, [zhanghm@nwpu.edu.cn](mailto:zhanghm@nwpu.edu.cn)

†Geer Teng, Chenhao Yang and Aojie Quan contributed to this paper equally.

Symbols used in the following text and the main manuscript are defined as follows in order of appearance.

| Symbol | Definition |
| --- | --- |
| $\boldsymbol{g}_{\boldsymbol{m}}$ | Equivalent conductance of the resonator |
| $\boldsymbol{Q}_{\boldsymbol{m}}$ | Mechanical Q factor |
| $\boldsymbol{\omega}_{\boldsymbol{0}}$ | Natural angular frequency of resonators |
| $\boldsymbol{T}_{\boldsymbol{ac}}$ | Temperature fluctuation |
| $\boldsymbol{v}_{\boldsymbol{ac}}$ | Input voltage |
| $\boldsymbol{X}_{\boldsymbol{th}}$, ${\dot{\boldsymbol{X}}}_{\boldsymbol{th}}$, ${\ddot{\boldsymbol{X}}}_{\boldsymbol{th}}$ | Displacement and first- and second- derivatives |
| $\boldsymbol{i}_{\boldsymbol{m}}$ | Output current |
| $\boldsymbol{s}$ | Laplace transform parameter |
| $\boldsymbol{R}_{\boldsymbol{th}}$ | Thermal resistance of the nanobeam |
| $\boldsymbol{C}_{\boldsymbol{th}}$ | Thermal capacitance of the nanobeam |
| $\boldsymbol{\alpha}$ | Thermal expansion coefficient of the nanobeam |
| $\boldsymbol{A}$ | Cross area of the nanobeam |
| $\boldsymbol{L}$ | Beam length |
| $\boldsymbol{E}$ | Young's modulus |
| $\boldsymbol{M}$ | Effective mass |
| $\boldsymbol{c}$ | Damping coefficient |
| $\boldsymbol{K}$ | Stiffness coefficient |
| $\boldsymbol{\pi}_{\boldsymbol{l}}$ | Piezoresistive coefficient |
| $\boldsymbol{I}_{\boldsymbol{dc}}$ | Direct current |
| $\boldsymbol{r}_{\boldsymbol{ac}}$ | Resistance fluctuation |
| $\boldsymbol{F}_{\boldsymbol{TE}}$ | Thermo-expansion force |
| $\boldsymbol{Q}_{\boldsymbol{eff}}$ | Effective quality factor |
| $\boldsymbol{\omega}_{\boldsymbol{f}}$ | Frequency in the de-embedded method |
| $\boldsymbol{\varphi}$ | Phase delay in the de-embedded method |
| a, b, $\boldsymbol{r}_{\boldsymbol{x}}$, $\boldsymbol{r}_{\boldsymbol{b}}$ | Quadrature amplitude of signals in the de-embedded method |
| $\boldsymbol{A}_{\boldsymbol{0}}$, $\boldsymbol{A}_{\boldsymbol{1}}$, $\boldsymbol{A}_{\boldsymbol{2}}$, $\boldsymbol{A}_{\boldsymbol{2}}$ | Amplitude of multi-harmonics |
| $\boldsymbol{F}_{\boldsymbol{1}}$, $\boldsymbol{F}_{\boldsymbol{2}}$ | Constants representing the components of thermo-expansion force |
| $\boldsymbol{\omega}$ | Actuation angular frequency |
| $\boldsymbol{f}_{\boldsymbol{d}}$, $\boldsymbol{2}\boldsymbol{f}_{\boldsymbol{d}}$, $\boldsymbol{3}\boldsymbol{f}_{\boldsymbol{d}}$ | Frequency of the harmonics |
| $\boldsymbol{K}_{\boldsymbol{2}}$ | Square stiffness nonlinear stiffness |
| $\boldsymbol{K}_{\boldsymbol{3}}$ | Duffing (cubic) nonlinear coefficient |
| $\boldsymbol{X}_{\boldsymbol{D}}$ | Peak value of amplitude |
| $\boldsymbol{\omega}_{\boldsymbol{r}}$ | Actual resonance angular frequency |
| $\boldsymbol{\rho}_{\boldsymbol{m}}$ | Density of silicon |

**Supplementary Note 1. The effective quality factor of thermal-piezoresistive tuning**

The nanobeam can be modelled as the parallel combination of its internal resistance R_A_ and the equivalent RLC circuit of the mechanical resonator, and the equivalent parameters are listed as follows:

| $R_{m}=\frac{1}{g_{m}}$ | (1) |
| --- | --- |
| $C_{m}=\frac{1}{{Q_{m}R}_{m}\omega_{0}}$ | (2) |
| $L_{m}=\frac{{Q_{m}R}_{m}}{\omega_{0}}$ | (3) |

Energy transformations between different physical domains can be described by the equivalent circuits as illustrated in Fig. 1(c) of the main manuscript, with three transfer functions:

| $H_{th}(s)=\frac{T_{ac}}{v_{ac}}=\frac{2I_{dc}R_{th}}{1+R_{th}C_{th}s}$ | (4) |
| --- | --- |
| $H_{m}\left( s \right)=\frac{X_{th}}{T_{ac}}=\frac{\alpha AE}{Ms^{2}+cs+K}$ | (5) |
| $H_{e}(s)=\frac{i_{m}}{X_{th}}=\frac{{\pi_{l}EI}_{dc}}{L}$ | (6) |

Considering $\omega_{0}=\sqrt{K/M}$ and $Q_{m}=M\omega_{0}/c$, the total transfer function between output current and the input voltage is:

| $\left. H_{total} \right\vert_{s=j\omega_{0}}=\frac{i_{m}}{v_{ac}}=\frac{2\alpha AE^{2}Q_{m}\pi_{l}R_{th}}{\left. jKL(1+R_{th}C_{th}j\omega_{0} \right)}{I_{dc}}^{2}$ | (7) |
| --- | --- |

Simplifying eq (7) with $K=EA/L$ and $\omega_{0}\gg\left( R_{th}C_{th} \right)^{-1}$, we have the equivalent conductance $g_{m}$:

| $\left\vert H_{total} \right\vert=g_{m}\approx\frac{2\alpha EQ_{m}\pi_{l}}{\omega_{0}C_{th}}{I_{dc}}^{2}$ | (8) |
| --- | --- |

Due to the thermal-piezoresistive principle, the input voltage and thermal-expansion force can be obtained:

| $v_{ac}=I_{dc}r_{ac}=I_{dc}E\pi_{l}R_{A}\frac{X_{th}}{L}$ | | (9) |
| --- | --- | --- |
| $F_{TE}(s)=\alpha T_{ac}EA=\alpha KE\pi_{l}R_{A}X_{th}\frac{2{I_{dc}}^{2}R_{th}}{1+R_{th}C_{th}s}$ | (10) | |

Equivalent to a mass-spring-damping system, the mechanical movements of the resonator can be expressed as:

| $\ddot{X}_{th}+\frac{c}{M}\dot{X}_{th}+\frac{K}{M}X_{th}=\frac{F_{TE}}{M}={I_{dc}}^{2}{\omega_{0}}^{2}\chi$ | (11) |
| --- | --- |
| $\chi=\frac{2\alpha E\pi_{l}R_{A}}{\left( \omega_{0}C_{th} \right)^{2}}(\frac{1}{R_{th}}-j\omega_{0}C_{th})$ | (12) |

where $X_{th}=x_{0}e^{j\omega_{0}t}$. Equation (11) can be reproduced as:

| $j{\omega_{0}}^{2}\left( \frac{1}{Q_{m}}-{I_{dc}}^{2}Im\left( \chi\right) \right)-{\omega_{0}}^{2}{I_{dc}}^{2}Re\left( \chi\right)=0$ | (13) |
| --- | --- |

then the equation can be derived from the imaginary part：

| $\frac{1}{Q_{eff}}=\frac{1}{Q_{m}}-{I_{dc}}^{2}Im\left( \chi\right)$, $Im\left( \chi\right)=-\frac{2\alpha E\pi_{l}R_{A}}{\omega_{0}C_{th}}$ | (14) |
| --- | --- |

The effective Q can thus be rewritten as:

| $Q_{eff}=\frac{Q_{m}}{1+R_{A}g_{m}}=\frac{Q_{m}}{1+\Upsilon I_{dc}^{2}}$, $\Upsilon=R_{A}\frac{2\alpha EQ_{m}\pi_{l}}{\omega_{0}C_{th}}$ | (15) |
| --- | --- |

As the piezoresistive coefficient $\pi_{l}$ is negative, there are specific values for $I_{dc}^{2}$ making $R_{A}g_{m}\subset[-1,0]$, pumping the effective quality factor.

**Supplementary Note 2. Principle of de-embedded method to eliminate feedthrough**

The de-embedded method provides a fast and direct path to extract basic information including resonance frequency and quality factor from recorded responses, applicable for the resonators with large motional impedances. The core of the method lies in the idea that to obtain basic parameters, the feedthrough signal has to be de-embedded from the measured responses first.

To be clearer, the equations of the output and the reference with $I_{dc}$, respectively, can be formulated as follows:

| $x=\mathrm{asin} \left( \omega_{f}t+\varphi\right)+bcos(\omega_{f}t+\varphi)$ | (16) |
| --- | --- |
| $r=r_{x}\sin\left( \omega_{f}t+\varphi\right)+r_{b}cos(\omega_{f}t+\varphi)$ | (17) |

where $\omega_{f}$ is the frequency, $\varphi$ the phase delay, a, b the quadrature amplitude of the extracted signal, the real and imaginary part in other words, $r_{x}$, $r_{b}$ the quadrature amplitude of the input reference signal. Subtracting the reference input in the quadrature components, the final amplitude of signal after feedthrough cancellation is represented as:

| $\left\vert x_{feed\_can} \right\vert=\sqrt{\left( a-r_{x} \right)^{2}+\left( b-r_{b} \right)^{2}}$ | (18) |
| --- | --- |

Then Amplitude-Frequency curves are gained, in Fig. 3(c) of the main manuscript.

**Supplementary Note 3. Resonators nonlinear motional model with multi-harmonics**

To research the amplitude-frequency property of resonators in resonance peak, we derive the relationship in the motion equation, with crucial parameters including thermal-expansion force and displacement fluctuation.

Basing on the equation (10) in linear model, the thermal-expansion force can be described as:

| $F_{TE}=\alpha KE\pi_{l}R_{A}X_{th}\frac{2{I_{dc}}^{2}R_{th}}{1+\left( \omega_{0}R_{th}C_{th} \right)^{2}}\left( 1-j\omega_{0}R_{th}C_{th} \right)\approx2\alpha KE\pi_{l}{I_{dc}}^{2}R_{A}R_{th}\frac{X_{th}}{\left( \omega_{0}R_{th}C_{th} \right)^{2}}\left( 1-j\omega_{0}R_{th}C_{th} \right)$ | (19) |
| --- | --- |

|   **Supplementary Fig. S1.** Frequency responses demodulated with the natural frequency signal, second- and third- order harmonics with various currents at the same voltage. The amplitude ($A_{1}$) of natural frequency component is a small amount, and the amplitudes of other waves ($A_{0}$, $A_{2}$ and $A_{3}$) are the infinitesimal of higher order. |
| --- |

Because multi-order harmonics are recorded in the output of the resonator, as shown in Fig. 5 of the main manuscript, we assume the displacement fluctuation with the actuation frequency $\omega$ as:

| $X_{th}=A_{0}+A_{1}\cos\omega t+A_{2}\cos2\omega t+A_{3}\cos3\omega t$ | (20) |
| --- | --- |

then derivatives and exponentiations are gained:

| $\dot{X}_{th}=-\omega(A_{1}\sin\omega t+2A_{2}\sin2\omega t+3A_{3}\sin3\omega t)$ | (21) |
| --- | --- |
| $\ddot{X}_{th}=-\omega^{2}(A_{1}\cos\omega t+4A_{2}\cos2\omega t+9A_{3}\cos3\omega t)$ | (22) |
| ${X_{th}}^{2}=\left( 2A_{0}A_{1}+A_{1}A_{2}+A_{2}A_{3} \right)\cos\omega t+\left( \frac{1}{2}{A_{1}}^{2}+2A_{0}A_{2}+A_{1}A_{3} \right)\cos2\omega t+\left( A_{1}A_{2}+2A_{0}A_{3} \right)\cos3\omega t+{A_{0}}^{2}+\frac{1}{2}{A_{1}}^{2}+\frac{1}{2}{A_{2}}^{2}$ | (23) |
| ${X_{th}}^{3}=\left( 3{A_{0}}^{2}A_{1}+3A_{0}A_{1}A_{2}+3A_{0}A_{2}A_{3}+\frac{3}{4}{A_{1}}^{3}+\frac{3}{4}{A_{1}}^{2}A_{3}+\frac{3}{2}A_{1}{A_{2}}^{2}+\frac{3}{2}A_{1}{A_{3}}^{2}+\frac{3}{4}{A_{2}}^{2}A_{3} \right)\cos\omega t+\left( 3{A_{0}}^{2}A_{2}+\frac{3}{2}A_{0}{A_{1}}^{2}+3A_{0}A_{1}A_{3}+\frac{3}{4}{A_{2}}^{3}+\frac{3}{2}{A_{1}}^{2}A_{2}+\frac{3}{2}A_{1}A_{2}A_{3}+\frac{3}{2}A_{2}{A_{3}}^{2} \right)\cos2\omega t+\left( 3{A_{0}}^{2}A_{3}+3A_{0}A_{1}A_{2}+\frac{1}{4}{A_{1}}^{3}+\frac{3}{4}{A_{3}}^{3}+\frac{3}{2}{A_{1}}^{2}A_{3}+\frac{3}{4}A_{1}{A_{2}}^{2}+\frac{3}{2}{A_{2}}^{2}A_{3} \right)\cos3\omega t+{A_{0}}^{3}+\frac{3}{2}A_{0}{A_{1}}^{2}+\frac{3}{2}A_{0}{A_{2}}^{2}+\frac{3}{2}A_{0}{A_{3}}^{2}+\frac{3}{4}{A_{1}}^{2}A_{2}+\frac{3}{2}A_{1}A_{2}A_{3}$ | (24) |

However, though the thermal-expansion force is in direct proportional to the displacement fluctuation, we regard it as the simple harmonic wave to simplify the model:

| $F_{TE}=\left( F_{1}+jF_{2} \right)\cos\omega t+\cdots\approx F_{1}\cos\omega t+F_{2}\sin\omega t$ | (25) |
| --- | --- |

where the imaginary unit $j$ is considered to change the phase of the force.

Different from the eq (11), we take the square and cubic stiffness nonlinearity into consideration in the motion equation:

| $M\ddot{X}_{th}+c\dot{X}_{th}+KX_{th}+K_{2}{X_{th}}^{2}+K_{3}{X_{th}}^{3}=F_{TE}=F_{1}\cos\omega t+F_{2}\sin\omega t$ | (26) |
| --- | --- |

then using the multi-harmonics balance method, the identical relations of constants, $\cos\omega t$ , $\cos2\omega t$ and $\cos3\omega t$ terms can be respectively written as:

| $KA_{0}+K_{2}{A_{0}}^{2}+K_{2}\frac{1}{2}{A_{1}}^{2}+K_{2}\frac{1}{2}{A_{2}}^{2}+K_{2}\frac{1}{2}{A_{3}}^{2}+K_{3}{A_{0}}^{3}+K_{3}\frac{3}{2}A_{0}{A_{1}}^{2}+K_{3}\frac{3}{2}A_{0}{A_{2}}^{2}+K_{3}\frac{3}{2}A_{0}{A_{3}}^{2}+\frac{3}{4}K_{3}{A_{1}}^{2}A_{2}+\frac{3}{2}K_{3}A_{1}A_{2}A_{3}=0$ | (27) |
| --- | --- |
| $-M\omega^{2}A_{1}+KA_{1}+K_{2}2A_{0}A_{1}+K_{2}A_{1}A_{2}+K_{2}A_{2}A_{3}+K_{3}3{A_{0}}^{2}A_{1}+K_{3}3A_{0}A_{1}A_{2}+K_{3}3A_{0}A_{2}A_{3}+K_{3}\frac{3}{4}{A_{1}}^{3}+K_{3}\frac{3}{4}{A_{1}}^{2}A_{3}+K_{3}\frac{3}{2}A_{1}{A_{2}}^{2}+K_{3}\frac{3}{2}A_{1}{A_{3}}^{2}+K_{3}\frac{3}{4}{A_{2}}^{2}A_{3}=F_{1}$ | (28) |
| $-4M\omega^{2}A_{2}+KA_{2}+K_{2}\frac{1}{2}{A_{1}}^{2}+K_{2}2A_{0}A_{2}+K_{2}A_{1}A_{3}+K_{3}3{A_{0}}^{2}A_{2}+K_{3}\frac{3}{2}A_{0}{A_{1}}^{2}+K_{3}3A_{0}A_{1}A_{3}+K_{3}\frac{3}{4}{A_{2}}^{3}+K_{3}\frac{3}{2}{A_{1}}^{2}A_{2}+K_{3}\frac{3}{2}A_{1}A_{2}A_{3}+K_{3}\frac{3}{2}A_{2}{A_{3}}^{2}=0$ | (29) |
| $-9M\omega^{2}A_{3}+KA_{3}+K_{2}A_{1}A_{2}+K_{2}2A_{0}A_{3}+K_{3}3{A_{0}}^{2}A_{3}+K_{3}3A_{0}A_{1}A_{2}+K_{3}\frac{1}{4}{A_{1}}^{3}+K_{3}\frac{3}{4}{A_{3}}^{3}+K_{3}\frac{3}{2}{A_{1}}^{2}A_{3}+K_{3}\frac{3}{4}A_{1}{A_{2}}^{2}+K_{3}\frac{3}{2}{A_{2}}^{2}A_{3}=0$ | (30) |

As derivations, linear and nonlinear coefficients, all contribute to the responses demodulated with frequency of $f_{d}$ and ${2f}_{d}$, causing the linear and nonlinear regimes. Also, due to $A_{2}\ll A_{1}$, the onset where nonlinearity generates of ${2f}_{d}$ is lower than that of $f_{d}$. And ignoring the high order infinitesimal $A_{3}$, there is only one term including the third-order harmonic, accompanied by the nonlinearity and the third-order harmonic signals appear only in nonlinear regime, same as experimental data in the Fig. 4 of the main manuscript.

Eliminating higher order infinite minor terms, $A_{0}$ can be gained from eq (27):

| $A_{0}=-\frac{1}{2}{A_{1}}^{2}K_{2}K^{-1}$ | (31) |
| --- | --- |

At the situation $K/M={\omega_{0}}^{2}=\omega^{2}$, the simplified equation of eq (28)~(30) can be rewritten as:

| $-\frac{{K_{2}}^{2}}{K}{A_{1}}^{3}+K_{2}A_{1}A_{2}+K_{2}A_{2}A_{3}+K_{3}\frac{3}{4}{A_{1}}^{3}=F_{1}$ | (32) |
| --- | --- |
| $-3KA_{2}+K_{2}\frac{1}{2}{A_{1}}^{2}-\frac{{K_{2}}^{2}}{K}{A_{1}}^{2}A_{2}+K_{2}A_{1}A_{3}=0$ | (33) |
| $-8KA_{3}+K_{2}A_{1}A_{2}-\frac{{K_{2}}^{2}}{K}{A_{1}}^{2}A_{3}+K_{3}\frac{1}{4}{A_{1}}^{3}=0$ | (34) |

then $A_{2}$ and $A_{3}$ can be described with $A_{1}$:

| $A_{2}=\frac{K_{2}{A_{1}}^{3}}{6K_{1}A_{1}-8F_{1}}$ | (35) |
| --- | --- |
| $A_{3}=\frac{((4{K_{2}}^{2}+6K_{1}K_{3})A_{1}-8K_{3}F_{1}){A_{1}}^{4}}{4(6KA_{1}-8F_{1})(8A_{1}K-9F_{1})}$ | (36) |

Because the quadratic sum of $\sin\omega t$ and $\cos\omega t$ equals to 1, the formula can be gotten:

| ${(KA_{1}-M{\omega^{2}A}_{1}-\frac{{K_{2}}^{2}}{K}{A_{1}}^{3}+\frac{K_{2}{A_{1}}^{4}}{6K_{1}A_{1}-8F_{1}}K_{2}+\frac{3}{128K}{A_{1}}^{4}{K_{3}}^{2}+\frac{3}{4}{A_{1}}^{3}K_{3})}^{2}+{(c\omega A_{1})}^{2}=F^{2}$ | (37) |
| --- | --- |

then the primary component $A_{1}$ can be expressed as:

| $A_{1}=\frac{F/M}{\sqrt{{(\frac{K}{M}-\omega^{2}-\frac{{K_{2}}^{2}{A_{1}}^{2}}{KM}+\frac{K_{2}{A_{1}}^{3}}{M(6K_{1}A_{1}-8F_{1})}K_{2}+\frac{3}{128KM}{A_{1}}^{3}{K_{3}}^{2}+\frac{3}{4M}{A_{1}}^{2}K_{3})}^{2}+{(\frac{c}{M}\omega)}^{2}}}$ | (38) |
| --- | --- |

The amplitude $A_{1}$ reaches the maximum when the following condition is satisfied:

| $\frac{K}{M}-\omega^{2}-\frac{{K_{2}}^{2}{A_{1}}^{2}}{KM}+\frac{K_{2}{A_{1}}^{3}}{M\left( 6K_{1}A_{1}-8F_{1} \right)}K_{2}+\frac{3}{128KM}{A_{1}}^{3}{K_{3}}^{2}+\frac{3}{4M}{A_{1}}^{2}K_{3}=0$ | (39) |
| --- | --- |

Due to the symmetry of the resonator, the terms with square nonlinearity are commonly ignored. Taking $K/M={\omega_{0}}^{2}$ into the eq (39):

| $\left( \frac{3{K_{3}}^{2}}{128K^{2}}A_{1}+\frac{3K_{3}}{4K} \right){A_{1}}^{2}=\frac{\omega^{2}-{\omega_{0}}^{2}}{{\omega_{0}}^{2}}\approx\frac{2(\omega-\omega_{0})}{\omega_{0}}$ | (40) |
| --- | --- |

Finally, the final approximate solution is gotten:

| $\frac{1}{2}(\frac{3{K_{3}}^{2}}{128K^{2}}A_{1}+\frac{3K_{3}}{4K}){X_{D}}^{2}\approx\frac{3K_{3}}{8K}{X_{D}}^{2}=\frac{\omega_{r}-\omega_{0}}{\omega_{0}}$ | (41) |
| --- | --- |

| 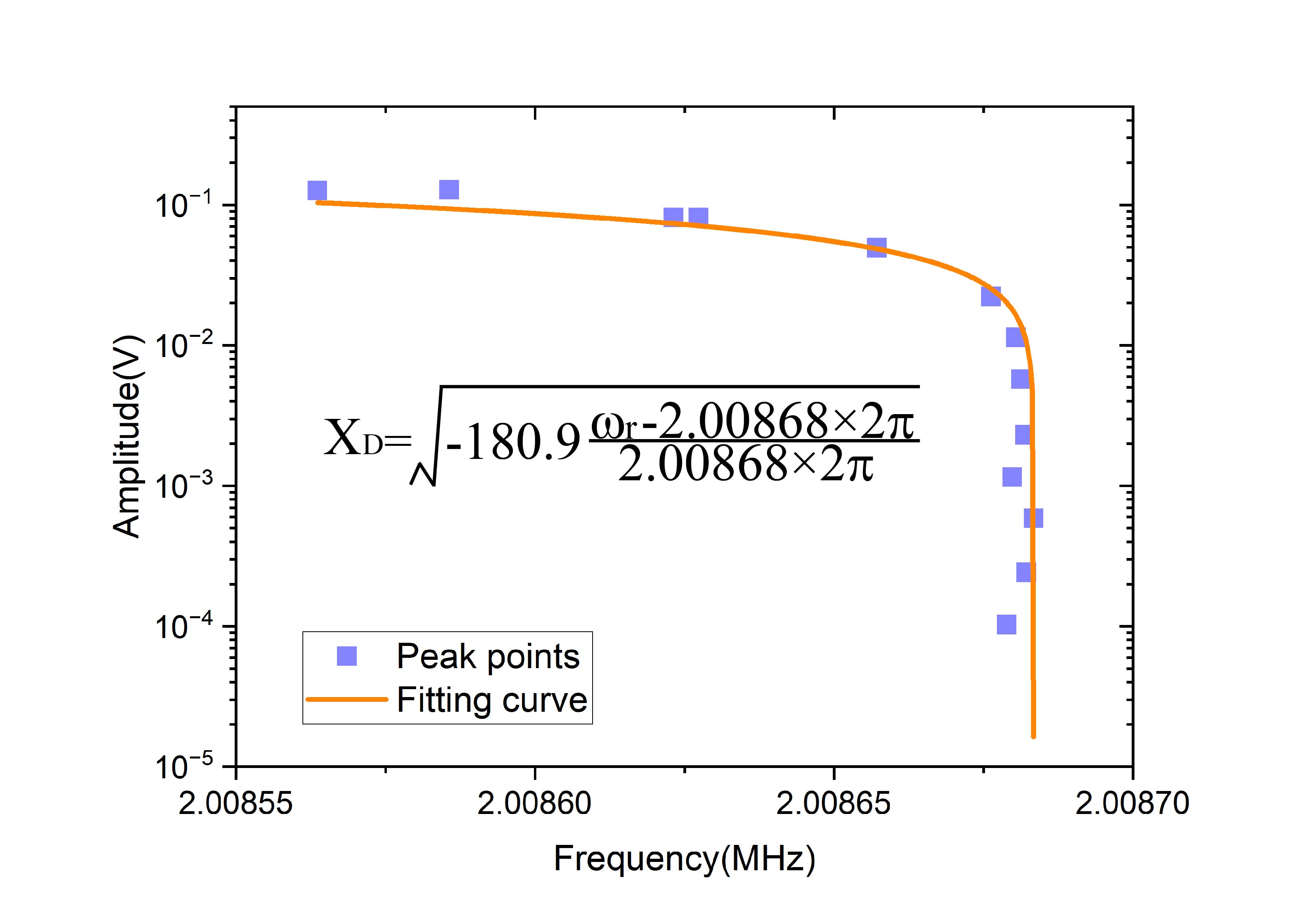  **Supplementary Fig. S2.:** The peak points of the natural frequency signal and the corresponding fitting curve. The chi-square of the fitting is demonstrated inferior to the limit of 10^-9^. The Duffing nonlinear coefficient $K_{3}$ is calculated at the basis of $K=M{\omega_{0}}^{2}$, and the following parameters: $E=169GPa, \rho_{m}$=3 $\text{g∙}\text{cm}^{\text{-3}}, M=\rho_{m}\times(14.8\mu m\times0.76\mu m+2\times240\mu m\times200\mu m+4\times100\mu m\times15\mu m)\times10\mu m$, $\omega_{0}=2\pi\times2.00868MHz$. |
| --- |
